# Analysis of the Relationship Between Microbial Community Succession and Volatile Flavor Compounds During Fermentation of Yunnan Traditional Rose Jam

**DOI:** 10.3390/foods15091590

**Published:** 2026-05-04

**Authors:** Jinping Zhou, Yanan Luo, Laifeng Chen, Yini Ma, Jijiang Dong, Dongmei Wang, Ghulam Mustafa, Shijun Li, Qiuye Lin, Zhenhui Cao

**Affiliations:** 1 College of Food Science and Technology, Yunnan Agricultural University, Kunming 650201, China; zhou_jinping101@163.com (J.Z.); lynmjysl@sina.cn (Y.L.); chenlaifeng2025@126.com (L.C.); edmund2348@gmail.com (Y.M.); 18787521189@163.com (J.D.); wangdongmei2025@126.com (D.W.); ghulammustafa9056@gmail.com (G.M.); lsj7783@126.com (S.L.); linqiuye@126.com (Q.L.); 2 School of Ethnic Medicine, Yunnan Minzu University, Kunming 650504, China; 3 Faculty of Animal Science and Technology, Yunnan Agricultural University, Kunming 650201, China

**Keywords:** microbial succession, flavoromics, aroma profile, high-throughput sequencing, volatile compounds

## Abstract

Yunnan traditional rose jam is made from fresh Rosa ‘Crimson Glory’ petals and brown sugar through natural fermentation and has been consumed by local people for years due to its characteristic floral aroma. The present study investigated the dynamics and correlations between the microbial communities and flavor quality during 98-day fermentation at ambient temperature (23.72 ± 1.26 °C). The total titratable acidity (0.20–0.29 g/100 g) and reducing sugars content (0.92–5.37 g/100 g) increased with fermentation, while the pH (4.30–5.47), total phenolics content (0.25–0.72 g/100 g), and total flavonoids content (0.13–0.32 g/100 g) decreased. High-throughput 16S rDNA and ITS sequencing revealed that *Klebsiella*, *Zygosaccharomyces*, *Starmerella*, and *Podosphaera* were the dominant microorganisms. Esters and terpenoids were the main flavor substances. Based on relative odor activity value evaluation, the relative levels of volatile compounds responsible for rose, sweet, and fruity odors increased during fermentation, while those responsible for a green character diminished. The physicochemical properties, especially the total titratable acidity and reducing sugars, as well as esters and terpenoids were positively related with *Zygosaccharomyces*. The present study systematically elucidated the correlation between microbial communities and flavor formation during fermentation of Yunnan traditional rose jam, providing key scientific basis for flavor-targeted regulation and standardized production of this traditional fermented food.

## 1. Introduction

Cultivation of edible roses is a long-standing tradition in the province of Yunnan in southwestern China. The varieties of edible rose plants cultivated in Yunnan are mainly *Rosa* ‘Dianhong’, followed by *Rosa* ‘Crimson Glory’, *Rosa* ‘Zizhi’ and *Rosa* ‘Jinbian’, and the planting area reaches over 4000 hectares [1]. Fermented rose jam is the most common rose-derived food and is classified as a traditional Chinese fermented food [2]. Rose jam retains the distinctive rich aroma of rose flowers and contains a variety of bioactive compounds, including polyphenols, flavonoids, polysaccharides, and essential amino acids [2,3]. Rose jam can be consumed directly or used as an ingredient in foods such as rose pastries, cookies, and sweet dumplings [4]. Yunnan traditional rose jam is produced by mixing fresh *Rosa* ‘Crimson Glory’ petals and brown sugar in a ratio of 2:3, followed by natural fermentation for a period of 3 to 6 months. In the rose jam from Pingyin, Shandong, as reported by Xia, et al. [2], *Rosa rugosa* ‘Plena’ petals and white sugar are combined in a 1:3 ratio and fermented at 30 °C for 50 days. Meanwhile, Uyghur traditional rose jam is prepared with rose petals and white sugar in a 1:3 mass ratio and fermented under sunlight for 5 to 6 months [5]. *Rosa* ‘Crimson Glory’ is characterized by its deep floral color, elegant morphology, and distinctive fragrance [6].

Fermentation duration is a key factor shaping the microbial community and flavor profile of fermented foods [7,8]. For instance, in light-flavor Xiaoqu Baijiu, the microbial community undergoes significant succession during an extended fermentation period of up to 98 days, with *Lactobacillus* eventually becoming overwhelmingly dominant and the complexity of flavor compounds increasing noticeably, especially the contents of esters and alcohols [9]. In addition, the concentration of sugar in the context of fermented food bases is another important influencing factor. Different sugar concentrations act as selection pressure, selecting the corresponding tolerant microorganisms, and thereby shaping the specific microbial community structure and ultimately influencing the formation of flavor. For example, varying sugar concentrations can not only alter the microbial community composition of kombucha (e.g., affecting the abundances of *Komagataeibacter* and *Brettanomyces*) [10], but also significantly influence its quality parameters, including the total acidity, total soluble solids, pH, viscosity, and color value [11]. Furthermore, research on rose jam quality evaluation indicates that samples from different regions exhibit similar distribution patterns in physicochemical properties, active components, and flavor substances, while significant regional differences exist across different production areas [3]. Based on the above evidence, the characteristics of rose jam vary with geographic region, sugar concentration, and fermentation duration, probably mediated by the selection of microbial communities. We assume that Yunnan rose jam may have a distinctive quality profile due to its different raw rose materials, sugar content and fermentation environment. Therefore, it is necessary to investigate the changes in the microbial composition and volatile compounds during the fermentation of Yunnan rose jam. Furthermore, although Xia et al. [2] have determined the microbial community, physicochemical properties, and flavor substance dynamics during the fermentation process of Pingyin rose jam, the relationship between microbial composition and key flavor formation remains unclear.

Therefore, the present study systematically investigated the multidimensional changes in Yunnan rose jam during a 98-day natural fermentation process. First, dynamic monitoring was conducted on key physicochemical indicators (pH, total acidity, reducing sugar, total phenolics, and total flavonoids) throughout fermentation. Secondly, headspace solid-phase microextraction coupled with gas chromatography–mass spectrometry (HS-SPME-GC-MS) was employed for the qualitative and quantitative analysis of volatile compounds. Combined with relative odor activity value (rOAV) evaluation, this approach elucidated the evolution of the flavor profile during fermentation. Meanwhile, 16S rDNA (bacteria) and ITS (fungi) sequencing techniques were used to reveal the composition and dynamic succession of dominant bacterial and fungal communities within the fermentation system. Furthermore, Spearman correlation analysis was applied to integrate the associations between the dominant microbial taxa and both the physicochemical indicators and the key characteristic flavor compounds, aiming to screen and identify the key microorganisms that may be related to flavor formation. These findings not only deepen the scientific understanding of the fermentation mechanism of this traditional food but, more importantly, provide crucial theoretical basis and data support for the targeted improvement of product flavor, process standardization, and quality enhancement through the regulation of microbial communities.

## 2. Materials and Methods

### 2.1. Chemicals and Reagents

The fFolin–Ciocalteu reagent, gallic acid, rutin, and D (+)-glucose were obtained from Beijing Solarbio Science & Technology Co., Ltd. (Beijing, China). The 2,2-diphenyl-1-picrylhydrazyl (DPPH) was sourced from TCI Shanghai Chemical Industry Development Co., Ltd. (Shanghai, China). Sodium hydroxide (0.1 mol/L) and iodine (0.05 mol/L) standard solutions were purchased from Codow, Guangzhou Howei Pharma Tech Co., Ltd. (Guangzhou, China). 3,5-dinitro salicylic acid (DNS) was obtained from Dalian Meilunbio Tech Co., Ltd. (Liaoning, China). The phenol, potassium sodium tartrate tetrahydrate, sodium sulfite, and sodium chloride (NaCl) were from Sinopharm Chemical Reagent Co., Ltd. (Shanghai, China). 3-hexanone-2,2,4,4-d4 was purchased from Merck (Darmstadt, Germany), and n-hexane from Sigma-Aldrich (St Louis, MO, USA). All remaining reagents were of analytical grade and sourced from China.

### 2.2. Preparation and Collection of Samples

Rosa ‘Crimson Glory’ were collected in Xiajinguanying, Hongta District, Yuxi City, Yunnan Province, China in 2021. The production method for the rose jam was based on the internal standards of Yunnan Yulongsheng Agricultural Technology Co., Ltd. (Yimen County, Yuxi City, Yunnan Province, China). Ultraviolet irradiation was regularly performed in the fermentation workshop. The specific process parameters were as follows: freshly harvested Yunnan Rosa ‘Crimson Glory’ petals (20 kg) were uniformly mixed with brown sugar (30 kg) in a sterilized mixer and placed into a sterilized food-grade polyethylene container with a capacity of 50 L. The container was sealed and the mixture was fermented at 23.72 ± 1.26 °C for 98 days. Three biological replicates were prepared. Samples were taken every 14 days for analysis. The samples were labeled according to their fermentation time as unfermented (D0) or fermented for 14 d (D14), 28 d (D28), 42 d (D42), 56 d (D56), 70 d (D70), 84 d (D84), or 98 d (D98). Before sampling, the rose jam was stirred thoroughly and 250 g of sample was collected from each container. Samples were divided into two aliquots: one was transported to the laboratory on ice for physical and chemical indicator testing, and the other was transported in liquid nitrogen to the laboratory for volatile compound detection and microbial sequencing.

### 2.3. Determination of Physicochemical Properties

#### 2.3.1. Determination of pH and Total Titratable Acidity

The pH value of rose jam was determined using a FE28 pH meter (Mettler Toledo Technology Co., Ltd., Shanghai, China). Total titratable acidity (TTA) was determined with titration to pH 7.0 with 0.1 mol/L NaOH and expressed as citric acid equivalents according to Chinese standard GB 12456-2021 National Food Safety Standard—Determination of Total Acid in Foods [12]. Briefly, 25 g of rose jam was mixed with 50 mL of carbon dioxide-free water and was then maintained at 80 °C in a DZKW-4 water bath (Beijing Zhongxing Weiye Instrument Co., Ltd., Beijing, China) for 30 min with shaking during maintenance. After cooling to room temperature, the mixture was diluted to 250 mL with carbon dioxide-free water and filtered through 201 fast quantitative filter paper (Hangzhou Specialty Paper Co., Ltd., Hangzhou, China). The filtrate is used for analysis.

#### 2.3.2. Determination of Total Reducing Sugar Content

Total reducing sugar content was determined using the DNS method [13]. Rose jam (2 g) was mixed with 1 mL of ultrapure water, ground to a paste in a JXFSTPRP-CL-BSC Cryogenic Grinder (Shanghai Jingxin Industrial Development Co., Ltd., Shanghai, China), and diluted to 100 mL in a volumetric flask. The diluted solution was allowed to stand for 30 min and was passed through 201 fast quantitative filter paper (Hangzhou Specialty Paper Co., Ltd., Hangzhou, China). The filtrate (1 mL) was mixed with 1 mL of water and 8 mL of DNS reagent (6.3 g DNS and 262 mL of 2 mol/L NaOH were added to 500 mL of hot water containing 185 g potassium sodium tartrate, then 5 g phenol and 5 g sodium sulfite were added, stirred, cooled, and diluted to 1000 mL with distilled water). The mixture was heated at 100 °C for 5 min, cooled to room temperature, and then diluted to 10 mL with distilled water. Absorbance was measured at 540 nm in a UV-5100 spectrophotometer (Shanghai Metash Instruments Co., Ltd., Shanghai, China) and the total reducing sugar concentration (g/100 g fresh weight) was calculated based on a glucose standard curve.

#### 2.3.3. Determination of Total Phenolic Content

Total phenolic content was determined using the Folin–Ciocalteu method [2]. Rose jam (2 g) was mixed with 10 mL of 75% ethanol solution and then ultrasonicated in an SB-5200DTD ultrasonic cleaner (Ningbo Scientz Biotechnology Co., Ltd., Ningbo, China) for 50 min at 70 °C, 40 kHz. The mixed solution was centrifuged at 2795× *g* in a Multifuge X1R laboratory centrifuge (Thermo Electron LED GmbH, Osterode am Harz, Germany) for 10 min to harvest the supernatant, which was then diluted with 75% ethanol. This diluted extract (0.5 mL) was mixed with 0.5 mL of Folin–Ciocalteu reagent and 1 mL 10% Na_2_CO_3_ solution. After incubating in the dark for 2 h, the absorbance at 765 nm was measured with a UV-5100 spectrophotometer (Shanghai Metash Instruments Co., Ltd., Shanghai, China). The standard of gallic acid was dissolved in 75% ethanol solution and diluted to 5 μg/mL, 10 μg/mL, 20 μg/mL, 30 μg/mL, 40 μg/mL, 50 μg/mL for analysis. The total phenolic concentration (g/100 g fresh weight) was calculated against the gallic acid standard curve.

#### 2.3.4. Determination of Total Flavonoid Content

The total flavonoid content in the rose jam extract was determined with the AlCl_3_ colorimetric assay as described by PérezHerrera, et al. [14] with minor modifications. Rose jam (2 g) was mixed with 75% ethanol and maintained at 70 °C in a DZKW-4 water bath (Beijing Zhongxing Weiye Instrument Co., Ltd., Beijing, China) for 1 h before cooling to room temperature for 30 min. The mixed solution was centrifuged at 2795× *g* in a Multifuge X1R laboratory centrifuge (Thermo Electron LED GmbH, Osterode am Harz, Germany) for 10 min to harvest the supernatant. Five-percent NaNO_2_ (0.5 mL) was added to 1 mL of the extract supernatant and left for 6 min. Then, 0.5 mL of 5% AlCl_3_ was added and, after a further 6 min, 5 mL of 4% NaOH. Absorbance was measured at 510 nm. The standard of rutin was dissolved in 75% ethanol solution and diluted to 2 μg/mL, 4 μg/mL, 8 μg/mL, 16 μg/mL, 24 μg/mL, 36 μg/mL, and 40 μg/mL levels for analysis and the total flavonoid concentration (g/100 g fresh weight) was calculated against a rutin standard curve.

### 2.4. Determination of Sample Color

The color of the rose jam was assessed using a CM-5 spectrocolorimeter (Konica Minolta Investment Ltd., Tokyo, Japan). The instrument was set with a D65 illuminant and a 2° observer angle. After instrument calibration was completed, the sample was placed in a solid sample dish. Three different areas of each sample were randomly measured sequentially for color determination. The parameters L*, a*, and b* were determined, with L* indicating lightness, a* redness (+) and greenness (−), and b* yellowness (+) and blueness (−). A large ∆E*ab value represents a substantial color change in a sample.

### 2.5. 16S rDNA and ITS Sequencing

Total microbial genomic DNA was extracted from rose jam samples using the TGuide 96S Magnetic Soil/Stool DNA Kit (Tiangen Biotech Co., Ltd., Beijing, China) according to the manufacturer’s instructions. The quality and concentration of DNA were determined using 1.8% agarose gel electrophoresis and a Synergy HTX microplate reader (BioTek., Winooski, VT, USA). The V3–V4 domains of the bacterial 16S rRNA and ITS1 rDNA regions of fungi were amplified using the following primer pairs and the Brilliant Lab 1000 automatic library building system (Biomarker Technologies, Beijing, China): 338F (5′-ACTCCTACGGGAGGCAGCA-3′) and 806R (5′-GGACTACHVGGGTWTCTAAT-3′) for 16S rRNA, and ITS1F (5′-CTTGGTCATTTAGAGGAAGTAA-3′) and ITS2R (5′-GCTGCGTTCTTCATCGATGC-3′) for ITS1. The cycling conditions for PCR amplification were as follows: initial denaturation at 95 °C for 5 min, followed by 25 cycles of denaturing at 95 °C for 30 s (1 min for ITS1), annealing at 50 °C for 30 s, extension at 72 °C for 45 s, single extension at 72 °C for 30 s (1 min for ITS1), and ending at 4 °C. The PCR products were purified, quantified, and homogenized to create libraries that were inspected, qualified, and sequenced using a NovaSeq 6000 system (Illumina, San Diego, CA, USA) according to the standard protocols in Beijing Biomarker Technologies Co., Ltd. (Beijing, China).

Regarding the sequencing results, for bacteria, a total of 1,849,105 paired-end reads were obtained from 24 samples. After quality control and assembly of the paired-end reads, 1,844,538 clean reads were generated, with each sample producing at least 47,597 clean reads and an average of 76,856 clean reads. As for fungi, a total of 1,919,488 paired-end reads were obtained from 24 samples. After quality control and assembly of the paired-end reads, 1,905,282 clean reads were generated, with each sample producing at least 79,075 clean reads and an average of 79,387 clean reads. The raw sequencing reads have been deposited in the NCBI Sequence Read Archive database (Accession Number: PRJNA1336344).

### 2.6. Sequence and Bioinformatics Processing

Trimmomatic (https://github.com/usadellab/Trimmomatic, v.0.33, accessed on 20 December 2022.) software was used to filter the sequenced raw reads. Cutadapt (http://cutadapt.readthedocs.org/, v.9.1) was used to identify and remove primer sequences, producing clean reads. The DADA2 method in QIIME2 (https://qiime2.org, v.2020.6) was used for de-noising of the data after quality control. Double-ended sequences were spliced and chimeric sequences were removed to produce the final data (non-chimeric reads). These sequences were clustered at the level of complete similarity and divided into different amplicon sequence variants (ASVs). Feature sequences were annotated using a naive Bayes classifier with a confidence level of 0.7. Each single sequence in an ASV was compared with the Silva bacterial (http://www.arb-silva.de) and Unite fungal (https://unite.ut.ee/) databases to obtain taxonomic information. QIIME2 (v.2020.6) was used to evaluate the alpha-diversity indices Chao1, ACE, Simpson, and Shannon and to carry out beta-diversity analysis to determine species diversity in the various samples.

### 2.7. Volatile Compound Analysis

HS-SPME-GC-MS (Agilent 8890 GC and 7000D MS system, Agilent Technologies, Inc., Santa Clara, CA, USA) was used for analysis of the volatile compounds as described by Zhang, et al. [15] with minor modifications. Rose jam (500 mg) was mixed with saturated NaCl solution to inhibit enzyme activity and 10 μL of 3-hexanone-2,2,4,4-d4 internal standard solution (50 μg/mL) was added. This mixture was placed in a headspace vial and heated at 60 °C for 5 min. A 120 µm DVB/CWR/PDMS fiber (Agilent) was then inserted into the vial and the sample was extracted at 60 °C for 15 min. Desorption of the volatile compounds captured on the SPME fiber was performed in the GC injection port at 250 °C for 5 min in non-split mode.

Identification and quantification of volatile compounds were performed with a 30 m × 0.25 mm × 0.25 μm DB-5MS (5% phenyl-polymethylsiloxane) capillary column. The carrier gas was helium at a linear velocity of 1.2 mL/min, the injector temperature was 250 °C, and the detector was set at 280 °C. The oven temperature program started at 40 °C (3.5 min), increasing at 10 °C/min to 100 °C, at 7 °C/min to 180 °C, at 25 °C/min to 280 °C, then held at 280 °C for 5 min. Mass spectra were acquired in electron impact (EI) ionization mode at 70 eV. The quadrupole mass detector, ion source, and transfer line temperatures were 150, 230, and 280 °C, respectively.

Analysis of the volatile compound data was conducted with the Metware cloud platform (https://cloud.metware.cn). Retention time and selected ion monitoring (SIM) of qualitative and quantitative ions were used to identify and quantify analytes using the Metware self-constructed high-coverage MS2 spectral tag library, using the ‘targeted spectra extraction’ algorithm, the mass spectrometry libraries of the National Institute of Standards and Technology (NIST17), surveys from the literature, and some standard compounds. Signals with aligned qualitative and quantitative ion peaks (same retention time range and similar peak shape) were accepted and their peak areas were integrated and manually adjusted [16]. Ions were analyzed sequentially based on their elution order. The quantitative ion for each compound was chosen to maximize quantitation accuracy. The chemical structures, names, and aromas of the volatile compounds were obtained from PubChem (https://pubchem.ncbi.nlm.nih.gov, accessed on 10 March 2023.) and the Good Scents Company Information System (http://www.thegoodscentscompany.com, accessed on 10 March 2023.). Sensory flavor characteristics of each compound were annotated and aroma attributes were assigned using reputable databases: Good Scents (http://www.thegoodscentscompany.com, accessed on 10 March 2023.), Food Flavor Lab (http://foodflavorlab.cn/#/home), and Perflavory Information System (http://perflavory.com) [17].

The relative content of the compounds in the sample was calculated as follows:

(1) $$C_{i}=\frac{V_{s}\times C_{s}}{M}\times \frac{I_{i}}{I_{s}}\times 10^{-3}$$

where *C_i_* represents the content of compound *i* in the sample (μg/g); *V_s_* is the volume of the internal standard added (μL); *C_s_* is the concentration of the internal standard (μg/mL); *M* is the mass of the sample (g); *Is* denotes the peak area of the internal standard; *I_i_* denotes the peak area of compound *i* in the sample.

The relative odor activity value (rOAV) was calculated as follows:

(2) $${rOAV}_{i}=\frac{C_{i}}{T_{i}}$$

where rOAV*_i_* is the relative odor activity value of compound *i*; *C_i_* is the relative content of the compound (μg/g); *T_i_* is the sensory threshold of the compound (μg/g).

### 2.8. Statistical Analysis

All analyses were conducted with three biological replicates. Data were expressed as mean ± standard deviation (*n* = 3) and plotted with GraphPad Prism (v.9). Results were analyzed with one-way analysis of variance (ANOVA) followed by Duncan’s *post hoc* test (*p* < 0.05) using IBM SPSS (v.20.0). Histograms of the microbial relative abundances were created with the BMKCloud (https://www.biocloud.net, accessed on 1 January 2020.). Redundancy analysis was performed using the Metware Cloud (https://cloud.metware.cn/, accessed on 29 December 2025.). Spearman correlation analysis (|r| ≥ 0.7 and *p* < 0.05) was conducted using R (v.4.4.3).

## 3. Results and Discussion

### 3.1. Analysis of Physicochemical Properties

**Figure 1:**
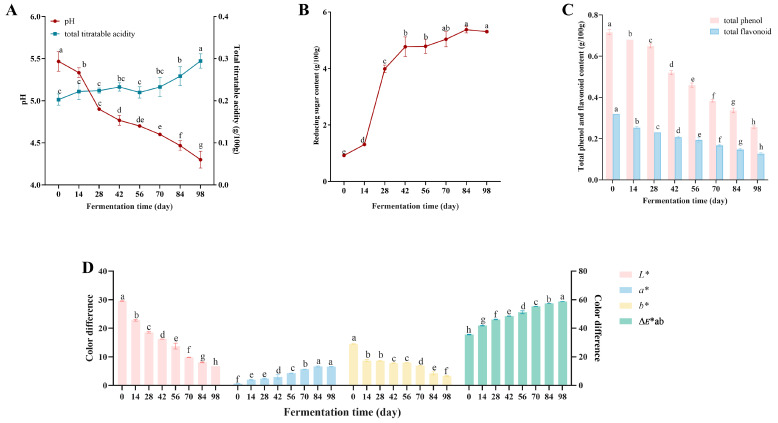
Physicochemical properties. (**A**) pH and total titratable acidity (TTA); (**B**) reducing sugars (RS); (**C**) total phenolics and total flavonoids; (**D**) color difference analysis. The results were expressed as mean values ± standard deviation. Different letters indicate significant differences among samples with different fermentation times. (*p* < 0.05).

Variations in pH and total titratable acidity (TTA) during fermentation of Yunnan rose jam are illustrated in Figure 1A. The pH decreased during fermentation, reaching 4.30 by the end. Correspondingly, TTA increased, peaking at 0.29 g/100 g fresh weight on day 98. Xia et al. [3] conducted a quality assessment of nine types of rose jam and found that the pH values of different rose jam samples varied, typically ranging from 3.78 to 4.90, which is consistent with our research results. These changes in pH and TTA may be related to the acid-producing microorganisms present during fermentation. For instance, inoculation with *Pediococcus pentosaceus* (isolated from rose jam) was found to increase the pH and TTA value of rose jam [18]. An increase in acidity can neutralize the sweetness of sugar [19]. As shown in Figure 1B, the total reducing sugar concentration increased during fermentation—sharply from 0.92 g/100 g fresh weight on day 0 to 4.77 g/100 g fresh weight on day 42, but more slowly from day 42 to day 98—reaching 5.37 g/100 g fresh weight. This increase may be due to the fact that sucrose (the main carbohydrate substrate) can be directly hydrolyzed by sucrose hydrolase into equal amounts of glucose and fructose [20].

The total phenolic and total flavonoid content decreased gradually during fermentation (Figure 1C), starting at 0.72 g/100 g fresh weight and 0.32 g/100 g fresh weight and reaching their lowest concentrations—0.25 g/100 g fresh weight and 0.13 g/100 g fresh weight, respectively—at day 98. It has been reported that the total phenols and total flavonoids in rose jam typically range from 2.18 to 13.48 mg/g and 1.83 to 10.89 mg/g, respectively [3]. Soluble phenolic compounds present during fermentation can be mobilized and degraded to small molecules, reducing the total phenolic content [21]. This process is related to the enzymes produced by the microbial community and lactic acid bacteria fermentation has been shown to reduce total phenolic and flavonoid levels in apple juice [22]. In addition, destruction of cell structures and non-enzymatic oxidation can also lead to a reduction in total phenolic and flavonoid concentrations [23]. Phenolic compounds and flavonoids are the main compounds responsible for the astringent taste in food [24,25]. Therefore, a decrease in the total content of phenols and flavonoids can alleviate the astringent taste of rose jam.

### 3.2. Analysis of Color Differences

Generally speaking, a uniform and bright rose color is the expected color for rose jam [26]. Changes in color were observed throughout the fermentation of Yunnan traditional rose jam (Figure 1D): L* and b* tended to decrease, while a* and ∆E*ab increased, indicating that the color intensified and changed to dark red, while the lightness decreased. These changes may result from enzymes produced during fermentation, such as polyphenol oxidase that catalyzes the conversion of polyphenols to quinones and is responsible for the appearance of brown pigments in damaged tissues [27]. Browning products resulting from the degradation of anthocyanins and other active compounds formed during fermentation (e.g., polymeric anthocyanins, tannins, and melanoidin pigments) may also have an impact [28].

### 3.3. Changes in Microbial Communities During Fermentation

#### 3.3.1. Bacterial and Fungal Diversity

The microbial genomic sequences present in the jam were screened to evaluate the microbial diversity during fermentation. Based on 100% similarity, 33,031 bacterial and 3152 fungal ASVs were identified. The alpha-diversity indices Ace, Chao1, Shannon, and Simpson reveal that the diversity and richness of the bacterial community were greater than that of the fungal community (Figures S1 and S2). Both the diversity and richness of bacteria increased during fermentation, unlike that of the fungi. The Ace, Chao1, and Shannon indices for bacteria fluctuated—initially increasing, then decreasing, and increasing again—in contrast to the fungi for which the opposite pattern was observed. In the study by Xia et al. [2], alpha-diversity index analysis during the fermentation of Pingyin rose jam indicated an increase in bacterial abundance and a decrease in fungal abundance, consistent with the findings of our study. The high-sugar environment in rose jam leads to osmotic pressure and molecular imbalance, which can inhibit the metabolism and growth of certain microorganisms [29]. On the other hand, an increase in sugar-tolerant bacteria (such as lactic acid bacteria) plays a significant role in driving fermentation [30].

#### 3.3.2. Dynamics of the Bacterial Community

**Figure 2:**
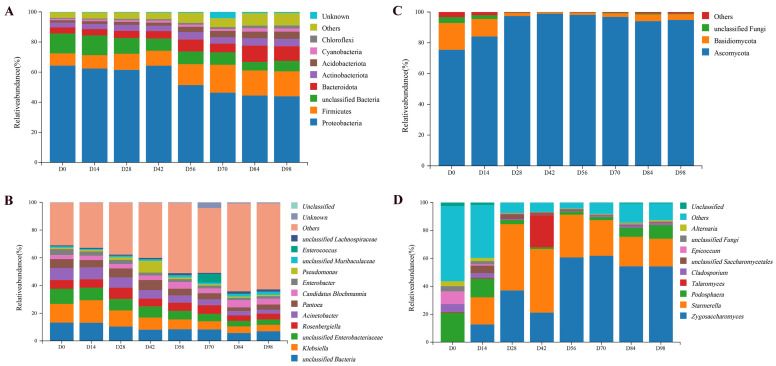
Composition of bacterial and fungal communities during fermentation. (**A**) Phylum level of bacteria; (**B**) genus level of bacteria; (**C**) phylum level of fungi; (**D**) genus level of fungi. D0–D98 indicate fermentation durations from 0 to 98 days.

Fifty bacterial phyla and 1962 genera were identified in Yunnan rose jam during fermentation (Figure 2). After 98 d, the dominant phyla were Proteobacteria, Firmicutes, Bacteroidetes, Actinobacteriota, Acidobacteria, Cyanobacteria, Choroflexi, and unclassified bacteria, each with a relative abundance > 1.00% (Figure 2A). Although the relative abundance of Proteobacteria decreased during fermentation (64.32% on day 0, 43.90% on day 98), it remained the most abundant phylum throughout. Liu, et al. [31] and Xia et al. [2] also reported that Proteobacteria predominated in a fermented rose jam. In addition, the relative abundance of unclassified bacteria also fell while other phyla increased. The dominant bacterial genera (relative abundance > 1%, Figure 2B) were unclassified bacteria, *Klebsiella*, unclassified *Enterobacteriaceae*, *Rosenbergiella*, *Acinetobacter*, *Pantoea*, *Candidatus blochmannia*, *Enterobacter*, *Pseudomonas*, unclassified *Muribaculaceae*, *Enterococcus*, and unclassified *Lachnospiracea*. Unclassified bacteria, *Klebsiella*, and unclassified *Enterobacteriaceae* were the most abundant, but they gradually decreased during fermentation. *Klebsiella* has been found in various fermented foods such as Pu-erh tea [32], soy sauce [33], and sausage [34]; due to its optimal growth pH value of 7.2, the acidic environment in rose jam may have affected its growth and reproduction [35]. Previous studies show that certain *Lactobacillus* strains can antagonize *Enterobacteriaceae* growth through the production of lactic acid [36] and other organic acids such as malic, formic, propionic, citric, and acetic acids [37]. The high abundance of *Rosenbergiella*, *Acinetobacter*, *Pantoea*, and *Pseudomonas* in rose jam is consistent with the findings of Xia et al. [2] and Liu, et al. [31]. It has been demonstrated that some *Acinetobacter* species and all *Rosenbergiella* species can tolerate high sugar concentrations (up to 60% *w*/*v*), which may explain the relatively high abundance of them during the fermentation of the rose jam [38]. The observed reduction in *Pantoea* during fermentation may be due to the falling pH [39]. *Pseudomonas*, which increased before decreasing during rose jam fermentation, is found in various rose jams [31]. The relative abundances of *Candidatus blochmannia*, unclassified *Muribaculaceae*, and unclassified *Lachnospiraceae* increased during fermentation. It should be noted that the genus classified as ‘Others’ (relative abundance < 1%) increased during fermentation and accounted for 62.03% at the end—more than half of the total bacterial population. These findings indicated that while the relative abundance of these species was low, the bacterial diversity increased considerably during the fermentation of the rose jam.

#### 3.3.3. Dynamics of the Fungal Community

Ten fungal phyla and 454 genera were identified during the fermentation of rose jam (Figure 2). Figure 2C shows the fungal phyla with relative abundances exceeding 1.00%. Ascomycota (75.45–94.85%) dominated, followed by Basidiomycota (0.85–17.41%) and unclassified fungi (0.13–3.80%). This is consistent with the findings of Xia et al. [2]. Liu, et al. [31] also reported that Ascomycota predominated (followed by unclassified fungi and Basidiomycota) in rose jams produced in other areas. After the 98 d fermentation of Yunnan traditional rose jam in the present study, Ascomycota abundance had increased, while Basidiomycota and unclassified fungi decreased.

The dominant fungal genera (relative abundance > 1%, Figure 2D) were *Zygosaccharomyces*, *Starmerella*, *Podosphaera*, *Talaromyces*, *Cladosporium*, unclassified *Saccharomyces*, *Epicoccum*, unclassified fungi, and *Alternaria*. The abundance of *Zygosaccharomyces* was extremely low (almost undetectable) as fermentation began, but its relative abundance increased as fermentation progressed and it became the predominant genus (60.67%) by day 56 and until the end. This is consistent with *Zygosaccharomyces* being a dominant fungal genera in rose jam from Dali, Yunnan Province [31], and the fact that these species are particularly tolerant of high-sugar conditions [40]. The relative abundances of *Starmerella* and *Talaromyces* increased then decreased, peaking at 45.55% and 23.01%, respectively, by day 42. *Podosphaera* exhibited the opposite pattern, being most abundant (21.14%) in the unfermented rose jam. Punyauppa-Path, et al. [41] have demonstrated that *Starmerella* was a dominant genus widely distributed in fermented foods, including high-sugar foods such as honey [42]. *Talaromyces* is a widespread genus that has been isolated from soil, plants, sponges, and foods [43]. It has been reported to produce a variety of secondary metabolites with antifungal, antimicrobial, and antitumor properties, including steroids, terpenoids, polyketides, and isocoumarins [44]. The relative abundances of *Cladosporium* and unclassified *Saccharomycetales* fluctuated during rose jam fermentation: *Cladosporium* initially decreased, then increased and finally decreased, while unclassified *Saccharomycetales* exhibited the opposite pattern. *Cladosporium* spp. are cosmopolitan in their distribution and are frequently isolated from soil, food, paint, textiles, and other organic matter [45]. *Saccharomycetales* is also one of the primary fermenters of *Miang*, a traditional fermented tea [46]. *Epicoccum*, unclassified fungi, and *Alternaria* abundances fell during fermentation of rose jam in the present study. *Zygosaccharomyces*, unclassified fungi, *Botrytis*, *Diaporthe*, and *Thermomyces* have been identified as dominant genera in rose jams produced in other areas [31], while *Alternaria*, *Podosphaera*, *Botryotinia*, *Cladosporium*, and *Aspergillus* are the main fungal genera present in rose jam made with *Rosa rugosa* cv. *Plena* [2]. These reports suggest that the composition of the fungal community varies slightly between different rose jams, but that *Zygosaccharomyces*, *Podosphaera*, *Talaromyces*, *Cladosporium*, and *Alternaria* are important in the formation of quality traits.

Compared with Pingyin rose jam (made from *Rosa rugosa* cv. Plena with white sugar fermentation), the microbial community characteristics of Yunnan traditional rose jam exhibited significant differences. In terms of bacteria, the dominant bacteria in Pingyin rose jam were *Streptophyta*, *Pantoea*, *Rosenbergiella*, and *Pediococcus* [2]; whereas in Yunnan rose jam, in addition to *Pantoea* and *Rosenbergiella*, the dominant bacteria also included *Klebsiella* and *Acinetobacter*, along with a large number of unclassified bacteria and unclassified *Enterobacteriaceae*. Regarding fungi, Pingyin rose jam was dominated by molds (*Alternaria* > 35%), with extremely low yeast abundance (*Saccharomyces* at most only 1.02%) [2]; in contrast, Yunnan rose jam is dominated by yeasts (*Zygosaccharomyces*, *Starmerella*), with a relatively low abundance of molds (e.g., *Cladosporium*). These results indicate that processing conditions such as the raw brown sugar material, low temperature, and long-term sealed fermentation shape a unique yeast-dominated microbial ecology in Yunnan rose jam, distinct from the mold-dominated Pingyin rose jam type. Combined with the analysis by Liu, et al. [31] of nine commercial rose jam samples from five regions (Kushui Town, Gansu; Pingyin, Shandong; Guiyang, Guizhou; Dali, Yunnan; and Yinchuan, Ningxia), significant differences in microbial genus-level composition were observed among different samples, which may be related to variations in production processes, fermentation conditions, and microorganisms associated with the raw rose materials.

Notably, the presence of *Pseudomonas*, *Acinetobacter*, and *Enterobacteriaceae* (e.g., *Klebsiella*), as well as fungi such as *Alternaria* and *Cladosporium*, in rose jam may raise food safety concerns. The rapid pH drop (below 4.30) and high sugar content created acidic and hyperosmotic conditions that strongly inhibited the growth of these bacteria. Their relative abundance declined during the later fermentation stages. Moreover, Yunnan rose jam is typically processed with heating before consumption (e.g., as a filling in baked goods), which may further reduce the potential risk. In addition, DNA-based amplicon sequencing cannot distinguish between viable and non-viable microorganisms. Future studies may employ culture-dependent methods to assess microbial activity, thereby enabling a more comprehensive evaluation of the product’s microbiological safety.

It should be noted that a portion of the microbial sequences obtained during fermentation could not be reliably assigned to known genera and were therefore classified as ‘unclassified’ or ‘unknown’. This observation reveals the current limitation of public reference databases (e.g., SILVA, UNITE) in resolving the microbial taxa endemic to traditional fermentation environments. Furthermore, genera with a relative abundance of less than 1% were merged into the ‘others’ category—a practice commonly adopted in microbiome analysis—which may, however, lead to the neglect of rare taxa with important ecological functions. Future research should employ metagenomic sequencing and targeted isolation to clarify the taxonomic affiliation of these unknown microbial components and to elucidate the potential functions of rare taxa.

### 3.4. Redundancy Analysis of Microorganisms and Quality Indicators

**Figure 3:**
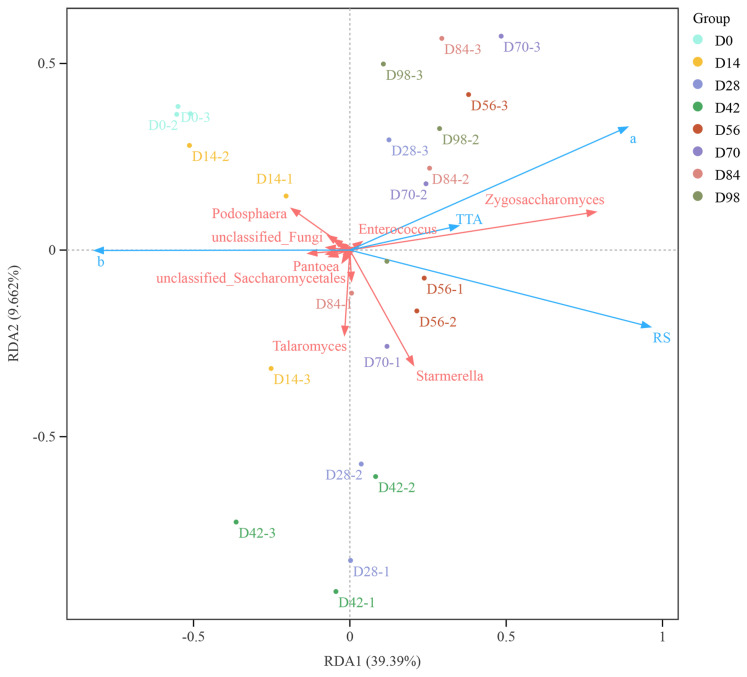
Redundancy analysis of the dominant microorganisms and quality indices. a and b: Color parameters of a* and b* values; TTA: total titratable acidity; RS: reducing sugars. Blue arrows indicate quality indices, and red arrows indicate dominant microorganisms. D0–D98 indicate fermentation durations from 0 to 98 days.

Redundancy analysis (RDA) is a linear model-based multivariate statistical method. It can simultaneously analyze the relationships between multiple microbial genera and multiple environmental factors. Through accounting for interactions among variables, RDA reveals complex correlations between microorganisms and environmental factors. The length of an environmental factor arrow represents the magnitude of its influence (explanatory power) on the distribution of the species data across the different sample groups. The angle between environmental factor arrows indicates the strength and direction of their correlation: acute angles represent positive correlations, while obtuse angles represent negative correlations. RDA of the dominant bacteria and fungi and the physiochemical indices are shown in Figure 3. The variables with variance inflation factor (VIF) > 10 were removed from the RDA. The RDA results showed that RDA1 and RDA2 explained 39.39% and 9.662% of the variance, respectively. Among the dominant microbial genera associated with fermentation parameters, *Zygosaccharomyces*, *Enterococcus*, and *Starmerella* indicated positive correlations with total titratable acidity (TTA), reducing sugars (RS), and a* value. Consistent with our observation, *Zygosaccharomyces lentus* can utilize sucrose as a source of fermentable sugar [47] and consequently increase reducing sugar content. *Enterococcus*, a member of lactic acid bacteria, can produce lactic acid from various sources, such as xylose, glucose, and fructose [48], and may contribute to the increase in TTA in Yunnan rose jam. Notably, *Pantoea*, unclassified Fungi, and *Podosphaera* were primarily positively correlated with b* value and those microbes combined with *Zygosaccharomyces*, *Enterococcus*, and *Starmerella* may be associated with the formation of the auburn red color of the Yunnan rose jam. In a study with douchi, Zhang, et al. [49] found that *Pantoea* was related to color differences in douchi, in accordance with our findings.

### 3.5. Changes in Volatile Compounds During Fermentation

#### 3.5.1. Dynamics of Differential Volatile Compounds

**Figure 4:**
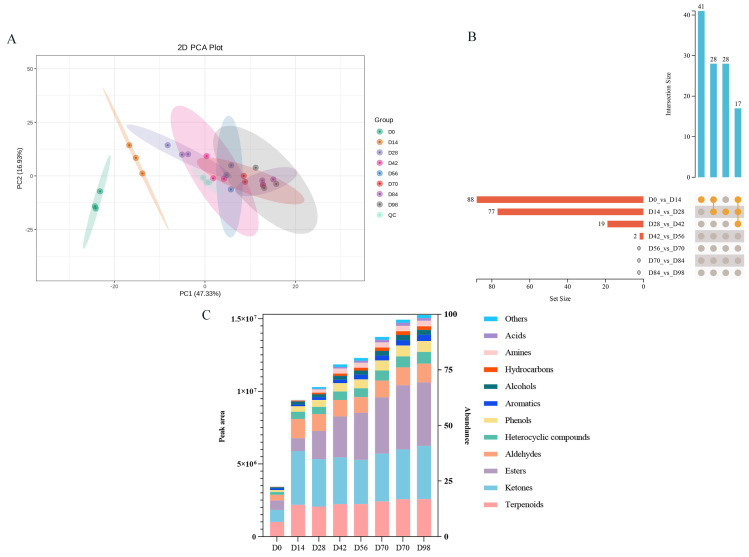
Metabolomic analysis of volatile compounds. (**A**) Principal component analysis; (**B**) UpSet diagram of the overlap of total differential metabolites in various pairwise comparisons; (**C**) peak area map of total differential metabolites. D0–D98 indicate fermentation durations from 0 to 98 days. QC means the quality control samples which were prepared by mixing 24 samples and were used to analyze the repeatability of samples under the same analysis method.

The flavor compounds in Yunnan traditional rose jam during fermentation were analyzed using HS-SPME-GC-MS. The principal component analysis (PCA) plot clearly revealed the dynamic changes in the volatile compound profiles of rose jam across different fermentation durations (Figure 4A). The first principal component (PC1), explaining 48.4% of the variance, served as the primary axis separating the samples, while the second principal component (PC2) accounted for 17.62%. The non-fermented group (D0) was distinctly separated from all fermented groups and located on the right side of the plot, indicating significant alterations of volatile compounds induced by fermentation. As fermentation time proceeded from 14 to 98 days, sample points shifted continuously along the PC1 axis from left to right, forming a clear time-dependent trajectory. Early fermentation groups (D14, D28) were positioned on the left, while mid- to late-stage groups (D42 to D98) progressively moved to the right, demonstrating ongoing and systematic changes in metabolite composition throughout the fermentation process. The confidence ellipse for each group is distributed sequentially with minimal overlap, further supporting distinct metabolic characteristics at different fermentation stages and good within-group reproducibility. The orthogonal partial least squares discriminant analysis (OPLS-DA) score plot for pairwise comparisons is shown in Figure S3. In the validation plot, orange and purple represent R^2^Y and Q^2^ of the permuted models, respectively, while the black arrows indicate the R^2^X, R^2^Y, and Q^2^ values of the original model. In an OPLS-DA model, R^2^X and R^2^Y denote the explained variances of the X matrix and Y matrix, respectively, and Q^2^ represents the predictive ability of the model. For all comparison groups, R^2^Y exceeded 0.9 and Q^2^ exceeded 0.6, indicating that the constructed models were appropriate and exhibited good predictive performance. Moreover, the R^2^Y and Q^2^ values of the permuted models in the validation plot were all lower than those of the original model, suggesting that the OPLS-DA model was not overfitted. These results demonstrated that samples in different comparison groups were stable, with clear separation of principal components, and that significant differences in metabolites existed between the comparison groups. In summary, the OPLS-DA model showed good stability and strong predictive ability, and it could be used for subsequent analyses.

Based on the variable importance in projection (VIP) values obtained from the OPLS-DA model, metabolites with significant differences between fermentation groups were preliminarily screened. A total of 118 differential metabolites were identified (VIP > 1, FC > 2 or <0.5, and *p* < 0.05) (Figure 4B). Eighty-eight were identified between the D0 and D14 groups, 77 between D14 and D28, 19 between D28 and D42, and 2 between D42 and D56. Thus, the number of differential metabolites diminished over time. It is notable that no additional differential metabolites were detected after day 56, suggesting that the volatile components tended to stabilize in the latter stages of fermentation. Based on peak areas, the main volatile compounds were terpenoids, ketones, and esters (Figure 4C); whereas in Pingyin rose jam, the contents of certain aldehydes, alcohols, and ketones gradually increase with fermentation time [2]. Analysis by Xia et al. [3] of eight commercially available rose jam samples from five regions (Kushui, Gansu; Pingyin, Shandong; Guiyang, Guizhou; Dali, Yunnan; and Yinchuan, Ningxia) showed that alcohols, esters, and aldehydes were common sources of rose jam flavor, but the volatile compositions varied significantly with different regions, while samples from the same region had similar compositions. For example, Pingyin rose jam was rich in ketones and aldehydes, while Yinchuan rose jam was dominated by esters [3]. Compared with the above two rose jam products, Yunnan rose jam was characterized by terpenoids also being listed as major volatile compounds—a finding that reveals the unique flavor composition of Yunnan rose jam that distinguishes it from other rose jams.

**Figure 5:**
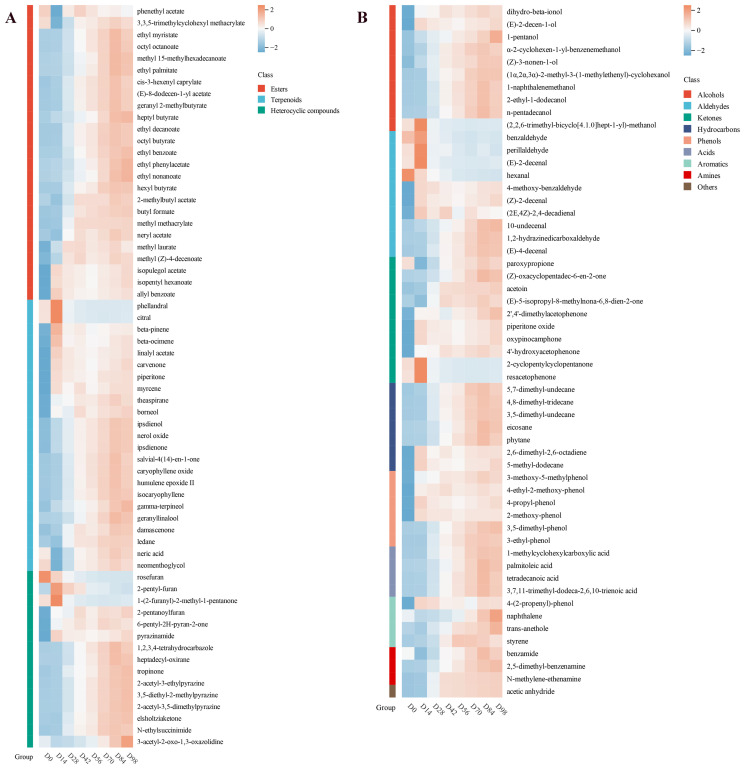
Heatmap of 118 differential metabolites, consisting of (**A**) 63 differential metabolites and (**B**) 55 differential metabolites. D0–D98 indicate fermentation durations from 0 to 98 days.

Changes in the 118 differential metabolites during fermentation (25 esters, 23 terpenoids, 15 heterocyclic compounds, 10 alcohols, 10 aldehydes, 10 ketones, 7 hydrocarbons, 6 phenols, 4 acids, 4 aromatics, 3 amines, and 1 other) are shown in Figure 5. The heatmap shows that the concentration of the vast majority of volatile metabolites increased during fermentation, while only a few decreased. Esters were the most numerous volatile components in Yunnan traditional rose jam. Microbial fatty acid and amino acid metabolisms have significant roles in ester biosynthesis [50]. Esters contribute substantially to the characteristic aromas of many fruits and flowers. While the sensory attributes of individual compounds vary, acetate esters typically exhibit fruity or flower-like aromas [51]. Five volatile acetate esters were identified in the Yunnan rose jam—2-methylbutyl acetate, neryl acetate, phenethyl acetate, ethyl phenylacetate, and isopulegol acetate—and we hypothesize that they significantly influence its flavor profile. The peak area map shows that terpenoids were the most abundant volatile component, providing a rich variety of pleasant scents ranging from floral to fruity, woody, and balsamic [52]. Terpenoids are derived from two common, interconvertible five-carbon (C_5_) precursors: isopentenyl diphosphate and its allylic isomer dimethylallyl diphosphate. In plants, these C_5_ precursors are synthesized via two distinct pathways: the mevalonate (MVA) pathway and the methylerythritol phosphate (MEP) pathway [53]. This abundance of terpenoids in the rose jam indicates that its pleasant aroma accumulates as fermentation proceeds. The third group of significant differential metabolites is the heterocyclic compounds. The Maillard reaction is common in food processing and storage and is influenced by many factors including protein source, hydrolysis conditions, molecular weight of polypeptides, temperature, and pH. It generates aldehydes, ketones, furans, pyrazines, and other heterocyclic compounds [54]. Significant levels of pyrazines and furans were also present in the rose jam. Furans contribute caramel and toasted aromas to the product [55], while pyrazines impart a nutty aroma and are associated with a positive sensory experience [54]. Other volatile compounds were also identified, albeit in lower concentrations. Thus, the fermentation of Yunnan traditional rose jam involves complex chemical transformations that contribute to its distinctive flavor profile.

#### 3.5.2. Flavoromic Analysis of Differential Volatile Compounds

**Figure 6:**
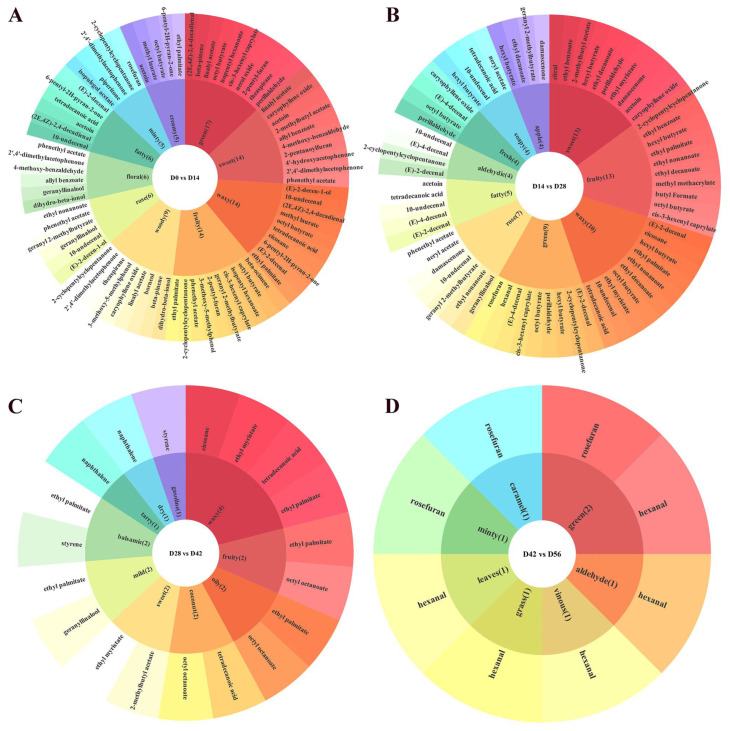
Flavor wheel illustrating the differential metabolites at different fermentation stages: (**A**) D0 vs. D14; (**B**) D14 vs. D28; (**C**) D28 vs. D42; (**D**) D42 vs. D56. D0–D98 indicate fermentation durations from 0 to 98 days.

To explore the changes in volatile compounds during the fermentation of the rose jam, a flavor annotation statistics analysis was performed on 118 differential volatile compounds. The top 10 sensory flavors with the highest number of annotated differential metabolites in each comparison group were selected for presentation (if more than 10 differential metabolites were annotated for a given sensory flavor, the top 10 metabolites with the highest VIP values were displayed) (Figure 6). Ultimately, 58 differential metabolites were identified (Table 1). The relative odor activity value (rOAV) was applied to further evaluate the contribution of these 58 metabolites to the flavor profile during the fermentation of rose jam.

Overall, the volatile profiling of Yunnan rose jam gradually became more complex with fermentation. At 14 days of fermentation, 29 flavor components with clear thresholds showed significant increases, among which (2E,4Z)-2,4-decadienal, 4-methoxybenzaldehyde, 2-pentyl-furan, (E)-2-decenal, and citral showed substantial increases, indicating their potential to be the major flavor compounds. These compounds exhibit flavor characteristics such as sweet, green, waxy, fruity, fatty, and floral. (2E,4Z)-2,4-decadienal is a type of aldehyde compound derived from fatty acids, and its content shows an increasing trend during black tea fermentation [56]. 4-methoxybenzaldehyde is also a key aroma component in acid whey fermented with *Ischnoderma benzoinum*, which contributes to a pleasant sweetish and marzipan-like odor [57]. 2-Pentyl-furan has a relatively low sensory threshold and significantly contributes to the aroma during fermentation of Chaling naturally fermented red Sufu [58]. (E)-2-decenal is also a basic volatile of aged fragrance in Qingzhuan tea [59]. Citral is a monoterpene aldehyde; its unique biological activities and lemon-like aroma have contributed to its widespread use in the food preservation industry [60]. After 28 days of fermentation of Yunnan rose jam, 22 flavor components with defined thresholds increased significantly. Ethyl decanoate, ethyl nonanoate, damascenone, trans-anethole, and phenethyl acetate—characterized by rose, sweet, fruity, waxy, oily, and apple notes—presented marked increases and may be considered the potential key flavor compounds at this stage. Ethyl decanoate and ethyl nonanoate provide a fruity flavor for fresh jujube brandy [61]. Damascenone is an important natural aroma compound and plays a crucial role in the flavor profile of wine during the fermentation and aging processes [62]. Trans-anethole, a terpenoid and a major constituent of essential oils derived from various medicinal plants, exhibits hypoglycemic and antioxidant activities [63].

There were seven flavor compounds with clear thresholds that significantly increased after 42 days of fermentation. Those were 2-methylbutyl acetate (fruity aroma), ethyl myristate (sweet aroma), ethyl palmitate (fruity aroma), styrene, and naphthalene. 2-Methylbutyl acetate is a prominent aroma-active compound commonly found in apples (contributing to their sweet, fruity flavor) and is synthesized via condensation of acetyl-CoA and 2-methyl butanol [64]. Ethyl myristate is commonly found in various alcoholic beverages and creates a pleasant aroma [65]. It accumulates during fermentation due to microbial metabolism and esterification reactions [66]. Ethyl palmitate has been shown to contribute to the fruity flavor of zhuhoujiang [67]. During fermentation, the relative contents of styrene and naphthalene gradually increased, but the sources of these compounds remained unclear. This may involve non-biological pathways (e.g., migration from food contact materials), and further experiments are needed to verify their formation mechanisms. Although both compounds were detected at low levels, long-term exposure may still pose potential health risks. Hence, optimizing fermentation conditions to minimize migration is necessary. Starting from day 56 of fermentation, the content of the volatile compounds tended to stabilize. For instance, the contents of hexanal and rosefuran significantly decreased continuously during the 0–56 day fermentation process and did not change afterwards. These two volatiles have green, leafy, and grassy notes and the reduction of their concentrations may decrease the above attributes of Yunnan rose jam. Overall, during the fermentation process, the concentrations of volatile components in Yunnan rose jam that possess the characteristics of rose aroma, sweetness, and fruity notes significantly increased, whereas compounds associated with the green flavor attribute decreased to form the desired flavor profile of Yunnan rose jam.

It should be noted that the quantification of volatile compounds in this study was semi-quantitative (relative to internal standards) rather than absolute. The odor threshold values used for rOAV calculation were primarily obtained from simple matrices (e.g., water, air) and may not fully represent the actual perception thresholds in the complex food system. Future studies will employ absolute quantitative methods to accurately determine the concentrations of key aroma compounds in Yunnan rose jam, and to establish their perception thresholds directly in the authentic food matrix, thereby enabling a more reliable assessment of their aroma contributions.

### 3.6. Relationship Between Microflora and Characteristic Aroma Compounds

**Figure 7:**
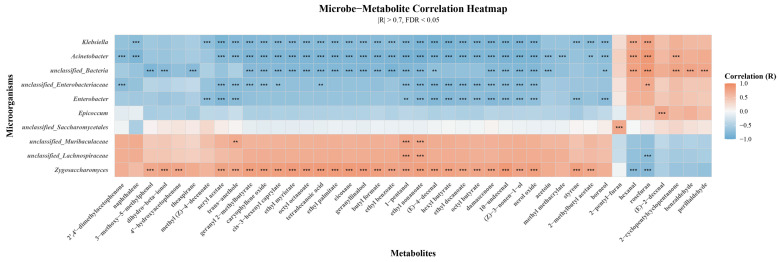
Spearman correlation analysis of the dominant microorganisms and characteristic aroma compounds. Spearman correlation coefficient ranges from −1 to 1; r < 0 indicates a negative correlation, r > 0 a positive correlation. Significant differences indicated by: ** FDR ≤ 0.01, *** FDR ≤ 0.001.

Spearman correlation was used to assess associations between characteristic aroma compounds and the dominant microorganisms, with FDR correction applied for multiple comparisons (Figure 7). Correlation analysis indicated that 28 characteristic aroma compounds were generally positively correlated with *Zygosaccharomyces*. These aroma compounds included 13 esters (2-methylbutyl acetate, butyl formate, cis-3-hexenyl caprylate, ethyl benzoate, ethyl decanoate, ethyl myristate, ethyl nonanoate, ethyl palmitate, geranyl 2-methylbutyrate, hexyl butyrate, neryl acetate, octyl butyrate, and octyl octanoate), 4 terpenoids (caryophyllene oxide, damascenone, geranyllinalool, and nerol oxide), 3 alcohols ((Z)-3-nonen-1-ol, 1-pentanol, and dihydro-beta-ionol), 2 aromatics (styrene and trans-anethole), 2 aldehydes ((E)-4-decenal and 10-undecenal), 1 ketone (4′-hydroxyacetophenone), 1 acid (tetradecanoic acid), 1 phenol (3-methoxy-5-methylphenol), and 1 hydrocarbon (eicosane). Previous studies have shown that the aroma-producing yeast *Zygosaccharomyces rouxii* is also be capable of fermenting sugars and converting various amino acids into alcohols [68]. Regarding ester synthesis, some non-conventional yeasts species possess alcohol acyltransferases (e.g., ATF, EHT1/EEB1, EAT1), which catalyze the condensation of acyl-CoA with alcohols to form esters [69]. For terpenoids, two mechanisms may be involved. Firstly, most yeasts synthesize terpenoids via the mevalonate (MVA) pathway, which sequentially converts acetyl-CoA into the terpenoid precursors isopentenyl pyrophosphate (IPP) and its isomer dimethylallyl pyrophosphate (DMAPP) through the action of ERG10, ERG13, HMG-CoA reductase, ERG12, ERG8, and MVD1 [70]. Secondly, yeasts can hydrolyze glycosidically bound terpenoid precursors via β-glucosidase to release free aromatic terpenoids [71,72]. Zhang, et al. [73] found that fermentation by non-*Saccharomyces cerevisiae* such as *Zygosaccharomyces. bailii*, *Hanseniaspora opuntiae*, and *Zygosaccharomyces. rouxii*) increased the concentrations of acids, ketones, esters, terpenes, and phenols in low-alcohol pear beverages. The above evidence suggests that *Zygosaccharomyces*, as a dominant fungal genus, may be related with the development of complex flavors during fermentation of Yunnan rose jam. Notably, the declines in rosefuran and hexanal were closely linked to reduced levels of specific bacterial genera, namely *Klebsiella*, *Acinetobacter*, and unclassified bacteria, indicating that they may be the indicator microorganisms correlated the green flavor and could be manipulated as a strategy to improve the aroma characteristics of Yunnan traditional rose jam.

## 4. Conclusions

This study systematically evaluated the correlation between the ecological succession of microbial communities and the formation of flavor metabolites during the natural fermentation of Yunnan traditional rose jam. The physicochemical indicators during fermentation altered, while TTA and RS content increased and pH and the levels of phenolics and flavonoids decreased. Based on the flavoromic analysis, it was determined that 4-methoxybenzaldehyde, ethyl decanoate, damascenone, and other compounds contributing to the unique sweet, fruity, and rose-like aromas of Yunnan rose jam increased significantly. The yeast *Zygosaccharomyces* may be associated with physicochemical fluctuations and flavor formation during fermentation. Further research is needed to explore how *Zygosaccharomyces* influences the biosynthesis of these aroma-active compounds with inoculation. A comprehensive sensory evaluation including a sensory panelist, electronic tongue and electronic nose will be needed for further study. In summary, this study reveals the intrinsic pattern of quality changes in traditional Yunnan rose jam from the perspective of microbial ecology and flavor profiling correlations, providing a theoretical basis for flavor regulation and process optimization of traditional fermented foods.

## Figures and Tables

**Table 1:** The pattern of change in the 58 differential metabolites.

Compounds	Calculate RI	Quantitative Ion/Qualitative Ion	Threshold	OAV								Type			
				D0	D14	D28	D42	D56	D70	D84	D98	D0 vs. D14	D14 vs. D28	D28 vs. D42	D42 vs. D56
**Ester**															
ethyl decanoate	1393.97	88/101	0.005	16.45	83.28	1691.78	2788.88	3298.11	4384.54	4677.89	4472.61	up	up	-	-
ethyl nonanoate	1294.87	88/101	0.01	0.84	26.17	128.83	227.51	295.33	477.11	589.73	660.13	up	up	-	-
methyl laurate	1523.15	74/87	0.0035	-	69.81	130.39	131.23	122.65	136.91	141.16	121.95	up	-	-	-
methyl (Z)-4-decenoate	1323.27	74/41	0.003	24.08	59.84	100.69	108.72	99.80	109.51	115.41	113.75	up	-	-	-
2-methylbutyl acetate	875.54	70/55	0.005	9.32	-	23.29	51.44	53.51	56.32	61.50	60.22	down	up	up	-
phenethyl acetate	1258.75	104/91	0.24959	57.53	29.93	60.24	65.65	63.72	62.50	60.30	57.95	down	up	-	-
ethyl myristate	1793.1	88/101	0.18	0.02	0.86	13.12	28.14	39.11	50.97	59.74	50.12	up	up	up	-
isopentyl hexanoate	1253.69	70/71	0.32	6.88	39.54	34.54	33.50	32.81	37.20	37.28	40.24	up	-	-	-
ethyl benzoate	1172.57	105/77	1.43	0.62	1.30	8.36	14.86	19.72	25.13	27.16	29.10	-	up	-	-
methyl methacrylate	716.57	41/69	0.2	0.78	1.74	12.77	19.34	20.03	21.41	20.71	21.54	-	up	-	-
butyl formate	720.54	56/41	0.37	0.69	1.45	8.34	13.59	15.44	17.13	17.21	18.00	-	up	-	-
ethyl palmitate	1990.69	88/101	2	0.01	0.20	3.37	7.32	11.19	15.71	19.05	17.64	up	up	up	-
hexyl butyrate	1195.74	89/71	0.203	0.09	0.19	2.78	4.43	5.04	7.27	7.51	7.55	-	up	-	-
octyl butyrate	1393.97	71/89	0.25	-	0.13	2.30	3.71	4.40	5.93	6.25	6.00	up	up	-	-
neryl acetate	1364.15	69/93	2	0.15	0.13	0.39	0.50	0.48	0.55	0.58	0.56	-	up	-	-
isopulegol acetate	1254.19	136/121	-	-	-	-	-	-	-	-	-	up	-	-	-
octyl octanoate	1793.1	55/57	-	-	-	-	-	-	-	-	-	up	up	up	-
allyl benzoate	1254.19	105/77	-	-	-	-	-	-	-	-	-	up	-	-	-
geranyl 2-methylbutyrate	1592.91	69/57	-	-	-	-	-	-	-	-	-	up	up	-	-
cis-3-hexenyl caprylate	1592.91	82/67	-	-	-	-	-	-	-	-	-	up	up	-	-
**Terpenoids**															
damascenone	1383.07	69/121	0.0015	147.31	318.22	735.36	986.33	961.10	1125.19	1255.31	1258.08	-	up	-	-
nerol oxide	1154.94	68/67	0.08	15.26	41.37	54.51	67.45	76.42	88.09	93.78	93.04	up	-	-	-
linalyl acetate	1254.19	93/80	1	11.27	50.59	42.18	40.44	40.93	44.87	44.31	46.80	up	-	-	-
beta-ocimene	1036.99	93/91	0.034	11.03	35.57	26.63	27.40	27.50	28.89	29.69	31.08	up	-	-	-
beta-pinene	990.98	93/41	0.14	10.91	32.13	23.71	24.57	23.59	25.39	25.32	25.85	up	-	-	-
citral	1271.42	69/41	0.1	38.32	104.81	25.11	19.76	19.07	18.28	18.46	19.85	up	down	-	-
borneol	1170.05	95/110	0.18	0.74	8.53	6.98	9.68	8.87	10.29	10.18	10.88	up	-	-	-
piperitone	1254.19	82/110	0.68	1.49	5.43	4.72	4.60	4.66	5.34	5.23	5.33	up	-	-	-
geranyllinalool	1990.69	69/41	-	-	-	-	-	-	-	-	-	up	up	up	-
theaspirane	1305.91	138/82	-	-	-	-	-	-	-	-	-	up	-	-	-
caryophyllene oxide	1592.91	109/41	0.41	-	0.19	1.05	1.66	2.13	2.59	2.87	2.58	up	up	-	-
**Aldehyde**															
(2E,4Z)-2,4-decadienal	1294.87	81/41	0.00007	-	6959.21	5199.21	6222.37	4177.93	6068.78	4987.12	4762.95	up	-	-	-
4-methoxybenzaldehyde	1254.19	135/136	0.0002	431.84	2457.70	2204.47	2012.56	2031.03	2205.74	2272.33	2335.29	up	-	-	-
10-undecenal	1294.35	41/55	0.0035	-	17.68	38.51	63.48	81.50	118.18	141.47	149.33	up	up	-	-
(E)-2-decenal	1271.42	83/41	0.005	130.70	361.64	84.25	65.00	63.12	60.54	58.46	69.08	up	down	-	-
(E)-4-decenal	1195.74	84/41	0.025	2.24	3.89	13.38	19.63	23.01	31.11	32.94	33.11	-	up	-	-
hexanal	792.38	44/56	0.005	609.66	350.68	156.77	104.31	45.59	40.57	26.69	19.18	down	down	-	down
perillaldehyde	1271.42	68/79	0.03	5.99	14.76	3.94	4.16	3.25	2.78	3.37	2.64	up	down	-	-
benzaldehyde	960.43	77/106	0.35	1.51	2.31	0.90	0.78	0.71	0.69	0.72	0.70	-	down	-	-
**Aromatics**															
trans-anethole	1289.16	148/147	0.057	71.13	66.89	125.19	154.68	210.63	244.82	235.94	292.83	-	up	-	-
styrene	887.91	104/103	0.0036	-	12.46	40.80	125.60	191.94	200.54	177.57	153.21	up	up	up	-
naphthalene	1179.62	128/129	0.05	2.35	-	0.50	1.35	2.65	5.19	7.73	10.80	down	up	up	-
4-(2-propenyl)-phenol	1254.19	134/133	-	-	-	-	-	-	-	-	-	up	-	-	-
**Heterocyclic compound**															
2-pentyl-furan	990.98	81/82	0.006	49.53	464.03	279.25	238.97	148.13	146.44	113.20	98.12	up	-	-	-
6-pentyl-2H-pyran-2-one	1450.7	95/82	0.15	0.84	3.52	3.67	3.93	3.85	4.56	4.20	4.09	up	-	-	-
rosefuran	1097	135/150	0.08	28.14	17.44	5.79	3.17	1.55	0.95	0.58	0.47	down	down	-	down
2-pentanoylfuran	1170.05	110/95	-	-	-	-	-	-	-	-	-	up	-	-	-
**Ketone**															
acetoin	716.57	45/43	0.011	-	6.74	53.81	80.86	87.40	95.12	91.05	98.44	up	up	-	-
4′-hydroxyacetophenone	1451.24	121/136	5.5	0.04	0.14	0.13	0.15	0.15	0.16	0.16	0.16	up	-	-	-
2′,4′-dimethylacetophenone	1254.7	133/105	-	-	-	-	-	-	-	-	-	up	-	-	-
2-cyclopentylcyclopentanone	1271.42	84/67	-	-	-	-	-	-	-	-	-	up	down	-	-
**Alcohol**															
1-pentanol	772.07	42/55	5	0.01	0.03	0.04	0.05	0.06	0.07	0.08	0.10	up	-	-	-
(Z)-3-nonen-1-ol	1154.94	55/68	-	-	-	-	-	-	-	-	-	up	-	-	-
(E)-2-decen-1-ol	1253.69	57/41	-	-	-	-	-	-	-	-	-	up	-	-	-
dihydro-beta-ionol	1451.24	123/93	-	-	-	-	-	-	-	-	-	up	-	-	-
**Acid**															
tetradecanoic acid	1793.1	73/60	10	-	0.00	0.04	0.09	0.12	0.15	0.18	0.15	up	up	up	-
**Hydrocarbons**															
eicosane	1990.69	57/71	10,000	0.00	0.00	0.00	0.00	0.00	0.00	0.00	0.00	up	up	up	-
**Phenol**															
3-methoxy-5-methylphenol	1322.75	138/109	-	-	-	-	-	-	-	-	-	up	-	-	-

Notes: rOAV: Relative odor activity value; D0–D98 indicate fermentation durations from 0 to 98 days; up indicates that the metabolite is upregulated in the comparison group; down indicates that the metabolite is downregulated in the comparison group; the symbol ‘-’ means not detected or not available.

## Data Availability

The original contributions presented in this study are included in the article/Supplementary Materials. Further inquiries can be directed to the corresponding author.

## Supplementary Materials

The following supporting information can be downloaded at https://www.mdpi.com/article/10.3390/foods15091590/s1, Figure S1. Analysis of bacterial community α diversity. (A) ASVs number; (B) ACE index; (C) Chao 1 index; (D) Simpson index; (E) Shannon index; (F) Coverage index. D0–D98 indicate fermentation durations from 0 to 98 days; the results were expressed as mean values ± standard deviation. Different letters indicate significant differences among samples with different fermentation times. (*p* < 0.05). Figure S2. Analysis of alpha diversity of fungal communities. (A) ASVs number; (B) ACE index; (C) Chao 1 index; (D) Simpson index; (E) Shannon index; (F) Coverage index. D0–D98 indicate fermentation durations from 0 to 98 days; the results were expressed as mean values ± standard deviation. Different letters indicate significant differences among samples with different fermentation times. (*p* < 0.05). Figure S3. Score plots and permutation validation plots of OPLS-DA for different comparison groups. A–G represent the score plots, and a-g represent the permutation validation plots. D0–D98 indicate fermentation durations from 0 to 98 days.
