# Supplementary material for: Analysis of the Relationship Between Microbial Community Succession and Volatile Flavor Compounds During Fermentation of Yunnan Traditional Rose Jam
Source: Foods. 2026 May 4;15(9):1590. doi: 10.3390/foods15091590 (PMC13164242; doi:10.3390/foods15091590)
Supplement: Supplementary file 1 [file foods-15-01590-s001.zip › foods-4257172-supplementary.pdf]

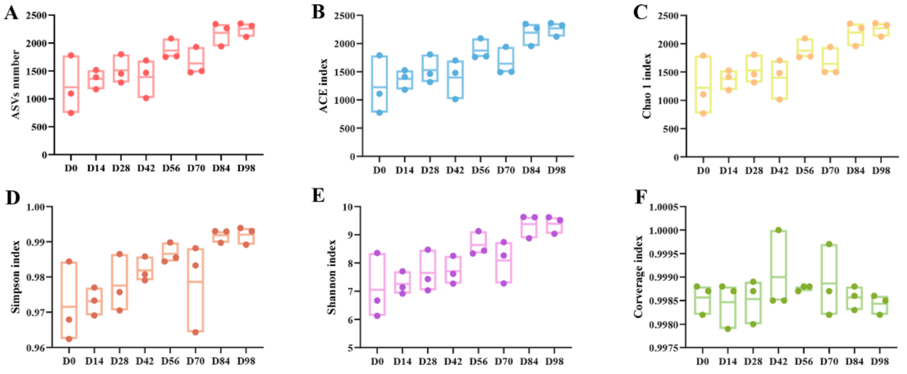

Figure S1. Analysis of bacterial community α diversity. (A) ASVs number; (B) ACE index; (C) Chao 1 index; (D) Simpson index; (E) Shannon index; (F) Coverage index. D0–D98 indicate fermentation durations from 0 to 98 days; the results were expressed as mean values ± standard deviation. Different letters indicate significant differences among samples with different fermentation times. ($p < 0.05$).

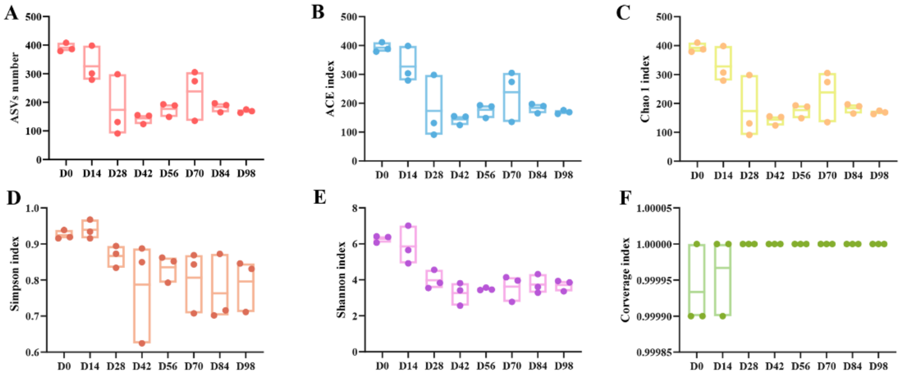

Figure S2. Analysis of alpha diversity of fungal communities. (A) ASVs number; (B) ACE index; (C) Chao 1 index; (D) Simpson index; (E) Shannon index; (F) Coverage index. D0–D98 indicate fermentation durations from 0 to 98 days; the results were expressed as mean values ± standard deviation. Different letters indicate significant differences among samples with different fermentation times. ($p < 0.05$).

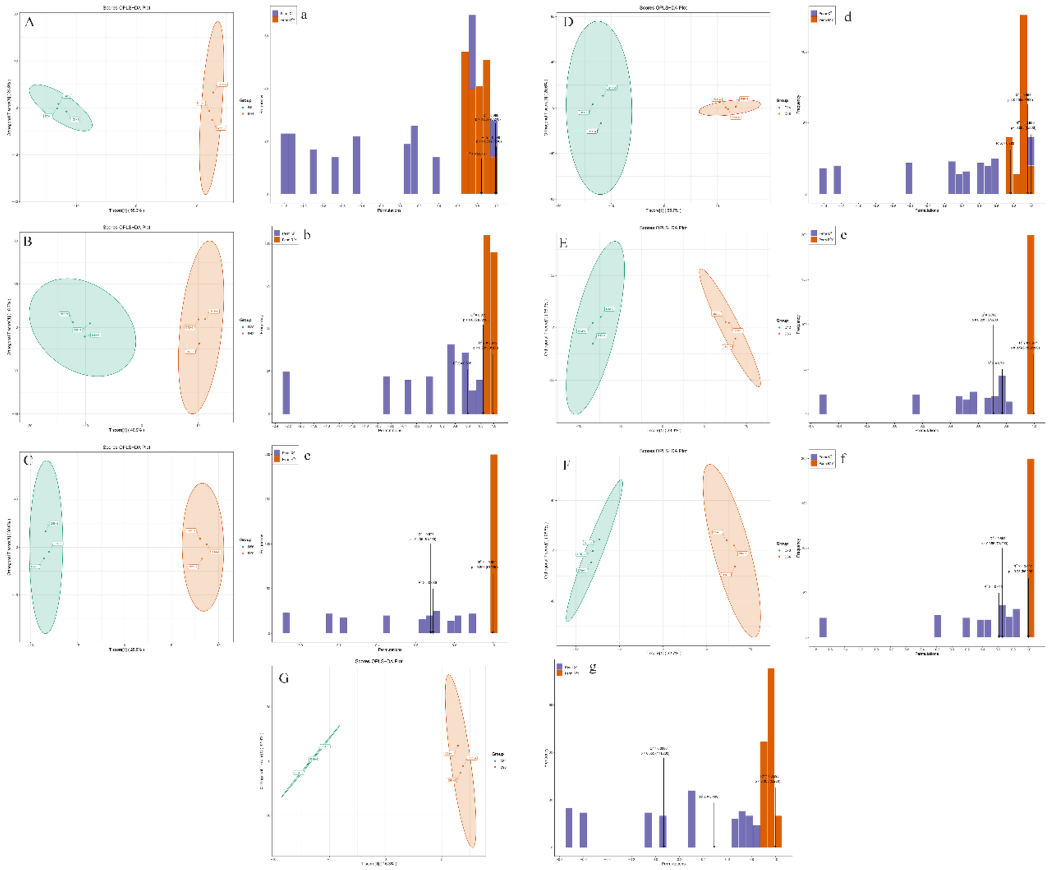

Figure S3. Score plots and permutation validation plots of OPLS-DA for different comparison groups. A–G represent the score plots, and a-g represent the permutation validation plots. D0–D98 indicate fermentation durations from 0 to 98 days.
